# SP prevents T2DM complications by immunomodulation

**DOI:** 10.1038/s41598-020-73994-1

**Published:** 2020-10-07

**Authors:** Sang-Min Baek, Kiyoung Kim, Suna Kim, Youngsook Son, Hyun Sook Hong, Seung-Young Yu

**Affiliations:** 1 College of Medicine/East-West Medical Research Institute, Kyung Hee University, Seoul, Republic of Korea; 2 Department of Biomedical Science and Technology, Graduate School, Kyung Hee University, Seoul, Republic of Korea; 3 Department of Ophthalmology, Kyung Hee University Hospital, Kyung Hee University, Seoul, Republic of Korea; 4 Department of Genetic Engineering, Graduate School of Biotechnology, College of Life Science, Kyung Hee University Global Campus, Yong In, Republic of Korea

**Keywords:** Endocrine system and metabolic diseases, Immunology, Stem cells, Diseases, Signs and symptoms

## Abstract

Type 2 diabetic mellitus (T2DM) is characterized by systemic inflammation and insulin resistance due to obesity, and this leads to critical complications, including retinopathy and nephropathy. This study explored the therapeutic effect of substance-p (SP), a neuropeptide, on T2DM progression and its complications. To examine whether SP affects glucose metabolism, lipid metabolism, systemic inflammation, and retinopathy, Otsuka Long-Evans Tokushima Fatty rats (OLETF, 27 weeks old) with chronic inflammation, obesity, and impaired bone marrow stem cell pool was selected. SP was intravenously injected and its effect was evaluated at 2 and 4 weeks after the SP injection. OLETF had typical symptoms of T2DM, including obesity, chronic inflammation, and poor glycemic control. However, SP treatment inhibited the body-weight gain and reduced circulating levels of free fatty acid, cholesterol, and triglyceride, ameliorating the obese environment. SP could suppress inflammation and rejuvenate bone marrow stem cell in OLETF rats. SP-mediated metabolic/immunological change could resolve hyperglycemia and insulin resistance. Histopathological analysis confirmed that SP treatment alleviated the dysfunction of target tissue with insulin resistance. OLETF rats have retinal damage from 27 weeks of age, which was reliably aggravated at 31 weeks. However, SP treatment could restore the damaged retina, sustaining its structure similarly to that of non-diabetic rats. In conclusion, systemic application of SP is capable contribute to the inhibition of the progression of T2DM and diabetic retinopathy.

## Introduction

Type 2 diabetes mellitus (T2DM) is a major disease that is characterized by hyperglycemia and hyperinsulinemia and the affects body’s metabolic activity. T2DM leads to many complications, including cardiac dysfunction, kidney damage, nephropathy, and retinopathy. The known risk factors of T2DM include obesity, aging, family history, and physical inactivity. T2DM comprises 90–95% of all cases of diabetes. The therapeutic options for T2DM include exercise, diet therapy, and pharmacotherapy. However, the multifactorial etiopathology of T2DM poses challenges to the effective management of T2DM. The main preventive measure for T2DM in the clinical setting is weight loss, which implicates the negative impact of obesity on the progression of T2DM. Obesity is caused by an excess of body fat that results from an imbalance between energy intake and energy expenditure^1^. Thus, obesity induces the elevation of free fatty acid (FFA) levels within the body, and this is a common phenomenon that is observed in T2DM^2^. High levels of FFA can block pancreatic β-cell function, thereby leading to diminished insulin secretion^3,4^ and reduced glucose consumption in the insulin-target tissues, including liver, skeletal muscle, and cardiac muscle^3^. Moreover, elevated FFA levels can enhance the expression of Toll-like receptor (TLR)-4 and TLR-2 to induce the activation of NF-KB and pro-inflammatory responses. The activation of inflammatory responses further stimulates the production of other pro-inflammatory cytokines, both locally and systemically, and those exacerbate the inflammation^1,5,6^. Besides the FFA-induced inflammation, obesity in itself represents a chronic inflammatory condition, as indicated by the high levels of interleukin 1 (IL-1), IL-6, C-reactive protein, and tumor necrosis factor alpha (TNF-α)^1,2^. This inflammatory stress induces resistance to hormones such as leptin, thereby enabling a vicious cycle in T2DM. Thus, inflammation is the key trigger for the development of diabetes.

The secretion of pro-inflammatory factors impairs stem cell activity. The pro-inflammatory environment in T2DM negatively affects the stemness of bone marrow stem cells, with a resultant decrease in the proliferation rate, clonogenic potential, and expression of specific markers and an increase in the doubling time^7,8^. Given the role of the bone marrow stem cell in tissue regeneration, the impairment of stem cell function in T2DM may be associated with the development of the diabetes-related complications.

Among the diabetic complications, diabetic retinopathy (DR) is leading cause of adult blindness and is the most common complication of diabetes. Diabetic condition induces damage in nonvascular retinal neurons and Mu¨ller glial cell with inflammatory stress. The development of DR can be affected depending on stage of diabetes and its severity. But certainly, inflammatory stress can aggravate DR progression.

Substance-P (SP) is a highly conserved, 11-amino acid neuropeptide that is secreted from sensory nerve endings and within various non-neural cells^9^. SP acts on neurokinin receptor 1 (NK-1R) with high affinity and signals via G-protein coupled receptor pathway. The anti-inflammatory action of SP suppresses inflammation in various diseases, including spinal cord injury, diabetic ulcers, and rheumatoid arthritis^10–17^. SP can block retinal inflammation and inhibit the development of proliferative vitreoretinopathy (PVR)^18^. Moreover, SP is capable of repopulating the stem cell pool in the bone marrow, thereby accelerating tissue regeneration^19^. These functions of SP were assumed to ameliorate diabetes-induced inflammation and, furthermore, block the progression of the diabetes-related complications.

This study aimed to explore the therapeutic effect of SP on T2DM-induced retinal degeneration in a preclinical model of T2DM. Otsuka Long-Evans Tokushima Fatty (OLETF) Rat has been employed as T2DM animal model. The Otsuka Long-Evans Tokushima fatty (OLETF) rat is a spontaneously diabetic rat with polyuria, polydipsia, mild obesity, hypertension and dyslipidemia. Between 12 and 20 weeks after birth, OLETF rats exhibit mild obesity and hyperinsulinemia with a late onset of hyperglycemia at 18 weeks of age^20^. It is also reported that by 30 weeks of age the animals develop renal dysfunction and glomerular damage, simulating the end-stage kidney disease that is seen in advanced human nephropathy^21^. Moreover, the progression of diabetic retinopathy in OLETF was evaluated^22,23^. Referring to previous reports, OLETF was evaluated to be suitable for type 2 diabetic preclinical model.

Using OLEFT, effect of SP was evaluated by examining the serum biochemical factors that are associated with inflammation, obesity, and glucose regulation. The target tissue histology, bone marrow stem cell pool, and retinal thickness were analyzed for changes over a time course.

## Materials and methods

### Experimental animals

This study used the Otsuka Long-Evans Tokushima Fatty (OLETF) rat and the Long-Evans Tokushima Otsuka (LETO) rat as the experimental diabetes and non-diabetes control animals, respectively^24,25^. The OLETF and LETO rats were purchased from the central experimental animal laboratory (Seoul, Korea). Prior to the experiments, the animals were maintained in a 12-h light/12-h dark illumination cycle in an animal holding room and allowed to acclimatize to the new environment. All animals received standard chow and water ad libitum. All animal studies were approved by the Ethical Committee for Experimental Animals and conducted in accordance with the Institutional Animal Care and Use guidelines of Kyung Hee University Hospital (approval number KHMC-IACUC-14-010). The body weight of the study animals was measured every week.

### Glucose tolerance test

For the glucose tolerance test (GTT), after 16-h overnight fasting, glucose (2 g/kg) was intraperitoneally injected, and blood was collected from the tail vein at 0 (fasting glucose level), 30, 60, 90, and 120 min. Plasma glucose levels were determined by the glucose oxidase method on a free blood glucometer with test strips (Accu-Check Active meter system; Roche Diagnostics Corporation, Indianapolis, IN, USA).

### Administration of SP and NK-1R antagonist

The SP (Sigma) was diluted in saline (JW Pharmaceutical, Seoul, Korea) immediately before use and administered intravenously twice a week for 4 weeks at a dose of 5 nmol/kg. In the control group, only the saline vehicle was administered. SP was injected to LETO or OLETF from postnatal 27 weeks. Neurokinin receptor-1 antagonist (10nmole/kg, RP67580, Tocris) was intravenously treated to rat 5 min before SP injection.

### Histological analysis

The rats were euthanized at 0, 2, and 4 weeks after the commencement of the bi-weekly SP injection. The retina, spleen, liver, muscle, fat, pancreas, and femur were harvested, isolated, and fixed in 3.7% paraformaldehyde (Sigma-Aldrich, St. Louis, MO, USA) for 1 day. The samples were processed with a TP1020 tissue processor (Leica Biosystems, Wetzlar, Germany) to make paraffin blocks, and 4.0-µm-thick sections were prepared. For the hematoxylin and eosin staining, the paraffin-sectioned samples were dehydrated in alcohol for 2 min. After washing with tap water, the nuclei were stained with hematoxylin (Sigma-Aldrich) for 2 min and washed again with tap water. To stain the cytoplasm, eosin Y (Sigma-Aldrich) was applied for 1 min and then washed off with tap water. Trichrome staining was undertaken with the NovaUltra™ Masson Trichrome Stain Kit (IHC World, Woodstock, MD, USA).

The immunohistochemical staining was undertaken in accordance with the manufacturer’s instructions for the VECTASTAIN ABC Kit (Vector Laboratories, Burlingame, CA, USA). Briefly, the hydrated samples were treated with sodium citrate for antigen retrieval. After washing in phosphate-buffered saline (PBS), the samples were treated with 0.5% H_2_O_2_ to block the activity of endogenous hydrogen peroxidase, and permeabilized with 0.3% Triton-X100. Nonspecific binding was blocked by incubating the samples with 1% normal horse serum for 1 h at room temperature. Primary antibodies against cleaved caspase-3 (1:100; Abcam, Cambridge, MA, USA), insulin (1: 100, Cell signaling technology, Danvers, MA, USA), and glial fibrillary acidic protein (GFAP; 1: 300; Abcam, Cambridge, MA, USA) were added. After three washes in PBS, the samples were incubated with a biotin-conjugated secondary antibody for 1 h at room temperature. Thereafter, the samples were washed again with PBS, a substrate solution was added for 1 h, and the samples were maintained at room temperature. To visualize the reactive area in the tissues, the samples were treated with NOVARED (Vector Laboratories), then counterstained for nuclei with hematoxylin (Vector Laboratories) for 2 min, and mounted in synthetic mountant™ (Thermo, Waltham, MA, USA).

### Enzyme-linked immunosorbent assay

We used enzyme-linked immunosorbent assay to measure the TNF-α, IL-10 (BioLegend Inc., San Diego, CA, USA), leptin, and adiponectin (R&D systems, Minneapolis, MN, USA) concentrations in serum samples in accordance with the manufacturer’s instructions. Briefly, all reagents, standard dilutions, and samples were prepared as directed. Subsequently, 100 µL each of the calibrator diluent was added to the nonspecific binding and zero standard (B0) wells. The remaining wells received 50 µL of the standard, control, or sample. Next, 50 µL of the primary antibody solution was added to each well, followed by the addition of 100 µL conjugate 2 h later. The wells were then incubated with 100 µL substrate solution. When the color of the solution changed to blue, the reaction was terminated, and the optical density was measured with the wavelength correction set to 540 or 570 nm by using the EMax Endpoint ELISA Microplate Reader (Molecular Devices, Sunnyvale, CA, USA).

### Colony-forming assay

The femur was isolated and bone marrow aspirates were obtained by flushing the medullary cavities with complete medium. After washing with PBS twice, the cell number was counted. Thereafter, 5 × 10^7^ of mononuclear cells were plated and cultured in MSCGM (Mesenchymal stem cell growth medium, Lonza, Basel, Swiss) for CFU-F and EGM-2 (Endothelial cell growth medium, Lonza, Basel, Swiss) for CFU-E, and 1 × 10^4^ of mononuclear cells were plated in the Methocult (Hematopoietic stem cell medium, Miltenyie Biotech, Bergisch Gladbach, Germany) for HSC-CFU for 10 days. Then, the colonies were counted.

### Biochemical analysis

The blood samples from the rats were collected and were centrifuged at 12,000 rpm for 10 min to separate the serum. The serum concentrations of total cholesterol, triglyceride, aspartate aminotransferase (AST), and alanine aminotransferase (ALT) were measured (Knotus Co, Gyeong-gi, Korea). The serum FFA level was determined by a quantification assay kit (Colorimetric, Abcam, Cambridge, MA, USA).

### Measurement of retinal thickness

Between 27 and 31 weeks of age, all rats were scanned by using Spectral domain Optical Coherence Tomography (SD-OCT, Heidelberg Engineering; OCT scan speed: 40,000 A-scans per second, axial resolution: 7 µm optical, 3.5 µm digital). The retinal nerve fiber layer (RNFL) circular scans centered at the optic disc were averaged automatically per B-scan to acquire the image at a constant distance from the optic disc. The eyes were dilated with topical tropicamide and wetted with methylcellulose (1%) eye drops, followed by placement of a coverslip and a double aspheric 60-D Volk lens (Volk Optical, Inc., Mentor, OH). The total retinal thickness (TRT) and RNFL thickness at three points were measured in a 2600-µm diameter circle that was centered on the optic disc by using the RNFL circular scan.

### Statistical analysis

All data are presented as the mean ± standard deviation (SD) of three independent experiments. *P*-values of less than 0.05 were considered statistically significant. All statistical analyses were conducted by an unpaired, two-tailed Student *t*-test.

## Results

### OLETF rats showed impaired glucose regulation with deficiency of stem cell pool in the bone marrow

T2DM is mainly characterized by weight gain and glucose dysregulation. The resultant environment induces diverse complications. In this study, we have focused on diabetic retinopathy (DR) as a critical complication. Diabetic retinopathy is developed in the late stage after diabetes onset and thus, may cause no symptoms at early phase. However, accumulating the early evidence for diabetes development can cause the development of the DR and therefore, we have attempted to ascertain the SP effect on diabetic condition and development of DR.

**Figure 1:**
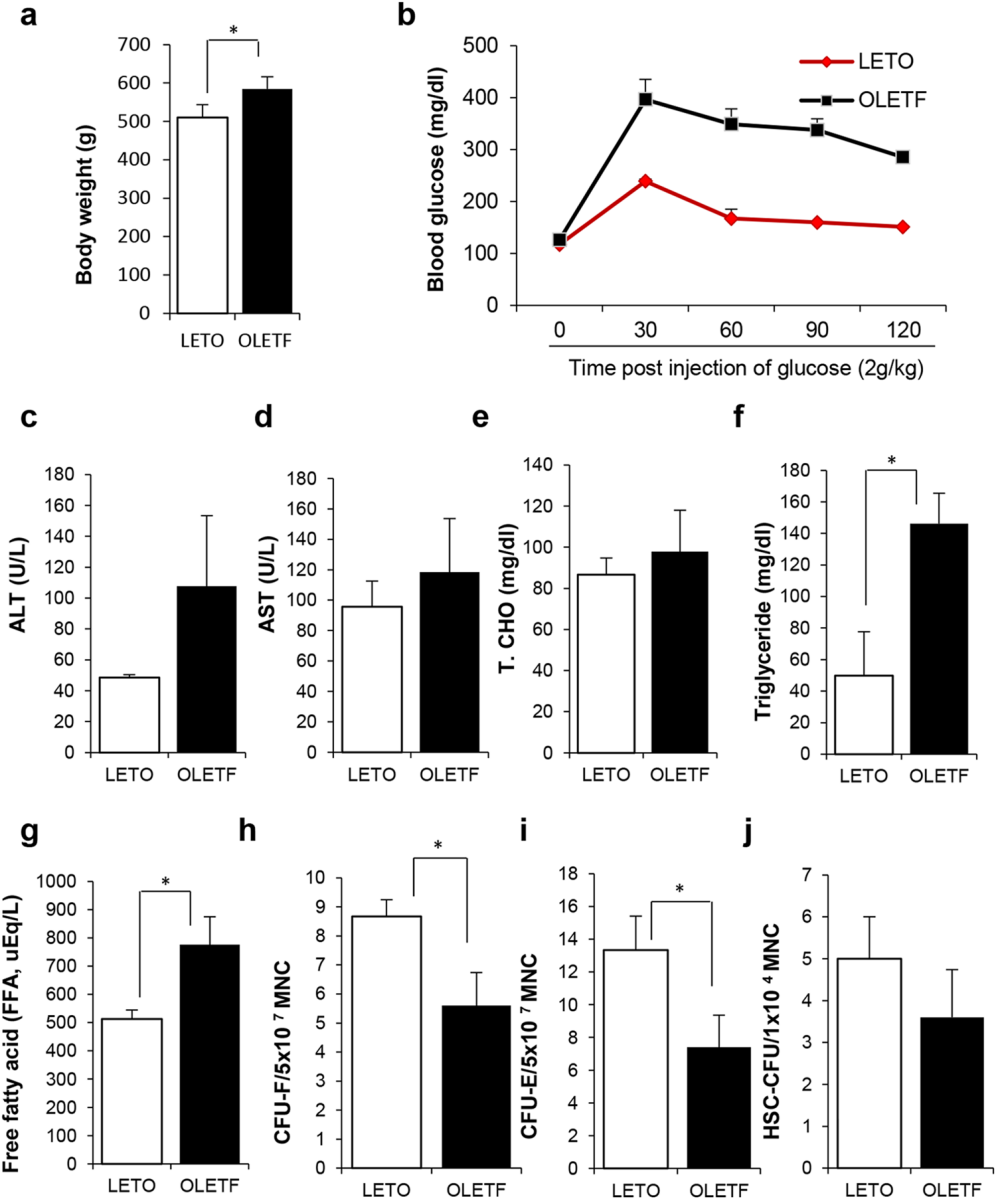
The examination for obesity-related biochemical makers and bone marrow stem cell pool of OLETF rat. (**a**), (**b**) Body weight and glucose tolerance test (GTT) in LETO and OLETF at postnatal 27 weeks were evaluated. (**c**)–(**g**) ALT, AST, total cholesterol, Triglyceride and free fatty acid were quantitatively determined. (**h**)–(**j**) Bone marrow stem cells from LETO and OLETF rats were assessed for colony-forming ability. CFU-F for MSC, (**h**) CFU-E for EPC (**i**) and HSC-CFU for HSC (**j**). *p* values of less than 0.05 were considered statistically significant (*p < 0.05, **p < 0.01, ***p < 0.001). N = 6/group.

Before the initiation of SP treatment, we confirmed the onset of diabetes by evaluating the body weight, blood glucose, and biochemical markers related to obesity in the OLETF rats at postnatal 27 weeks. Comparing to LETO control, body weight was higher in OLETF rats (Fig. 1a). Glucose tolerance test (GTT) analysis confirmed that OLETF rats have impaired glucose regulation (Fig. 1b). Both AST and ALT, which are indicators of hepatic damage, were elevated in OLETF rats. The total cholesterol, triglyceride, and FFA levels were clearly increased in OLETF, compared with LETO, rats (Fig. 1c–g). Neutral endopeptidase (NEP) is enzyme to degrade SP and its level is known to increase in diabetes. This was confirmed by this study. OLETF has higher NEP and lower SP, comparing to LETO (Supplementary Figure 1).

The diabetic environment can affect bone marrow stem cell function, and stem cells from diabetic patients demonstrate impaired activity in vitro^26^. In order to examine the stem cell pool in OLETF rats, we examined the colony-forming ability of bone marrow stem cell from LETO or OLETF rats at Postnatal Week 27. The CFU-F for mesenchymal stem cell (MSC), CFU-E for endothelial progenitor cell (EPC), and HSC-CFU for hematopoietic stem cell (HSC) were excessively suppressed in T2DM OLETF rats, compared with the LETO controls (Fig. 1h–j).

This result suggests that obese OLETF rats have failure of glucose control, accompanied by high FFA levels and reduced stem cell potential, thereby indicating the development of T2DM at 27 postnatal weeks in OLETF rats.

### Systemic injection of SP alleviates impaired glucose regulation in T2DM

The SP was intravenously injected to LETO or OLETF rats from 27 weeks for 4-week duration and, then, its efficacy was determined at 2 weeks (29 postnatal weeks) and 4 weeks (31 postnatal weeks) after SP treatment.

**Figure 2:**
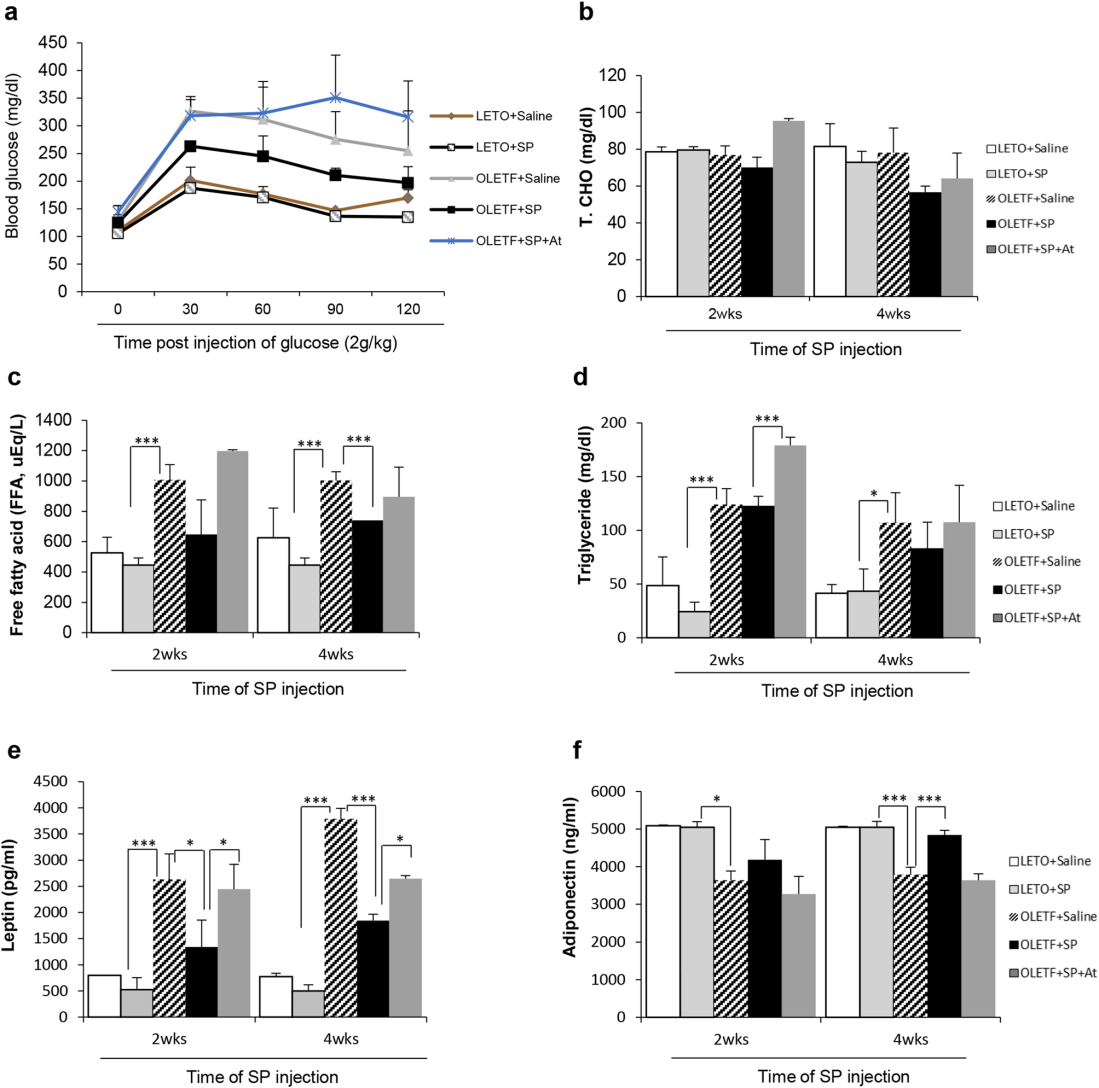
Substance P restores impaired glucose regulation by modulating serum obese/insulin resistance-related markers. Substance P (SP) was injected into LETO or OLETF rats from postnatal 27 weeks for 4 weeks; (**a**) results of the glucose tolerance test (GTT) in LETO and OLETF rats at postnatal 31 weeks were evaluated. (**b**)–(**f**) Serum biochemical and growth factors were quantified at 2 and 4 weeks post SP injection; *p*-values of less than 0.05 were considered statistically significant (*p < 0.05, **p < 0.01, ***p < 0.001). At: NK-1R antagonist. N = 10/group.

The SP injection for 2 weeks restored glucose regulation to some extent in OLETF rats, although its effect was non-significant (data not shown). However, SP treatment for 4 weeks markedly improved the glucose metabolic ability (Fig. 2a).

Serum biochemistry revealed that OLETF rats had elevated total cholesterol, FFA, and triglyceride levels, which were reduced by SP injection within 4 weeks (Fig. 2b–d). Leptin is important in food intake, and is involved in the activation of hepatic stellate cells in liver damage. OLETF rats had higher circulating leptin levels. Adiponectin, related to glucose/lipid metabolism and anti-inflammation, was deficient in OLETF rats, compared with the LETO controls. However, SP treatment reversed the OLETF-induced leptin/adiponectin profile by decreasing leptin and increasing adiponectin levels (Fig. 2e,f). This change of biochemical responses by SP might be related to the reduction in body-weight gain (Table 1).

**Table 1:** The effect of substance P on body weight in LETO and OLETF rats.

Post SP iv	LETO		OLETF	
	Saline	SP	Saline	SP
0 week	515 ± 23.2	516.7 ± 42.7	583.2 ± 28.1	585.5 ± 8.32
2 week	513.5 ± 17.3	505.6 ± 24.1	601.1 ± 22.1	591.4 ± 30.1
4 week	510.2 ± 17.4	495.1 ± 36.4	619.5 ± 11.0	590.6 ± 10.4

Body weight of LETO and OLETF. N = 12/group. Body weight was checked at 0, 2, and 4 weeks post SP injection in LETO and OLETF.

The effect of SP was observed at 2 weeks after commencement of the injections, and its effect undeniably became distinct 4 weeks after treatment initiation, corresponding to 31 postnatal weeks of age. All SP effects were blocked by co-treatment with the NK-1R antagonist (At).

These data suggest that SP treatment ameliorates the phenomenon observed in T2DM, to maintain a physiological status similar to that of LETO rats.

### SP treatment contributes to the restoration of pancreatic function

**Figure 3:**
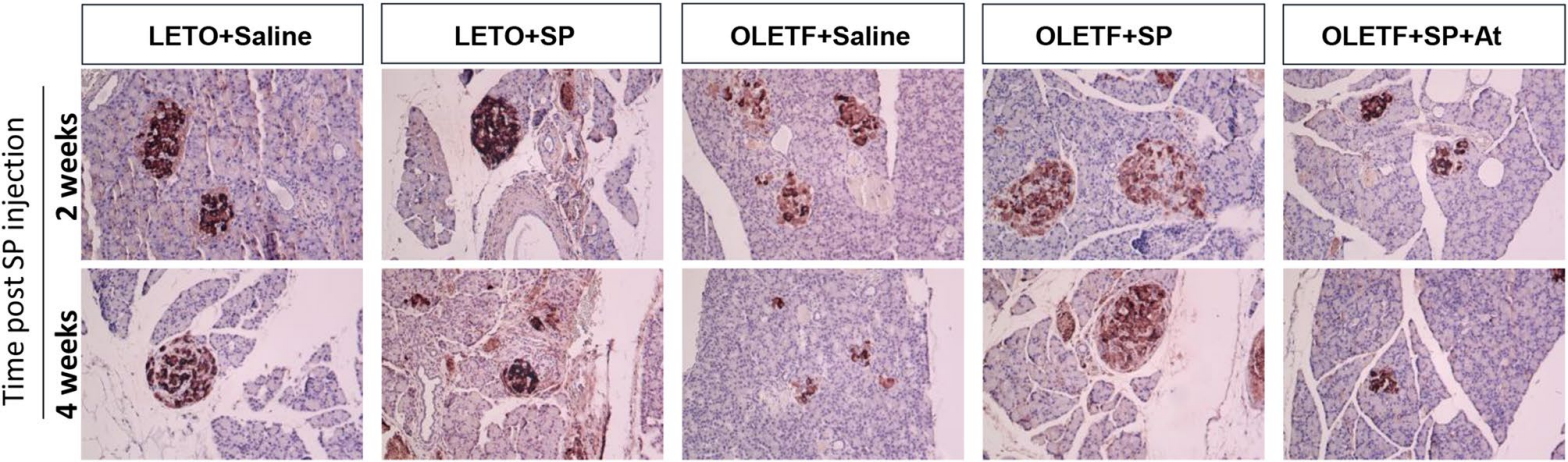
SP reduced the deficiency of insulin in beta cell of pancreas. Pancreas was fixed at 2 an d 4 weeks after SP injection in OLETF and then, histological analysis was conducted for insulin in beta cell of pancreas from LETO or OLETF. At: NK-1R antagonist. N = 10/group.

T2DM induces chronic insulin resistance, which increases FFA levels that is lipotoxic to the pancreatic β-cells^27^. This study proved that SP treatment could restore glucose regulation and decrease circulating FFA levels in OLETF rats (Fig. 2). This finding implies the possibility of the recovery of pancreas function by SP treatment. In order to check the effect of SP on the pancreas, insulin production was determined by immunohistochemical analysis (Fig. 3).

In LETO rats, insulin-positive β cells were mostly observed and their level was rarely changed by SP injection. In contrast, OLETF rats had destroyed islets and few β-cells that were positive for insulin. This was aggravated at Postnatal Week 31. Interestingly, SP injection increased the insulin-positive area and maintained a more intact shape of the pancreatic β-cell, compared with that of the saline-injected OLETF rats. SP effect was suppressed by NK-1R antagonist treatment.

This indicates that SP injection could resolve the insulin deficiency in the pancreas. Therefore, SP treatment might contribute to glucose regulation by enriching insulin in OLETF rats.

### SP repress hepatic damages and adipocyte hypertrophy

T2DM has insulin resistance in the liver, muscle, and fatty tissue. The diabetic condition induces pathological changes in these tissues to make them insulin resistant. Therefore, the inhibition of fatty change in the liver and muscle or suppression of adipocyte dysfunction is a crucial target to relieve diabetes progression. We found that SP injection blocked pancreatic β-cell destruction (Fig. 3). Next, we checked whether SP treatment affects target tissues that directly control glucose level.

**Figure 4:**
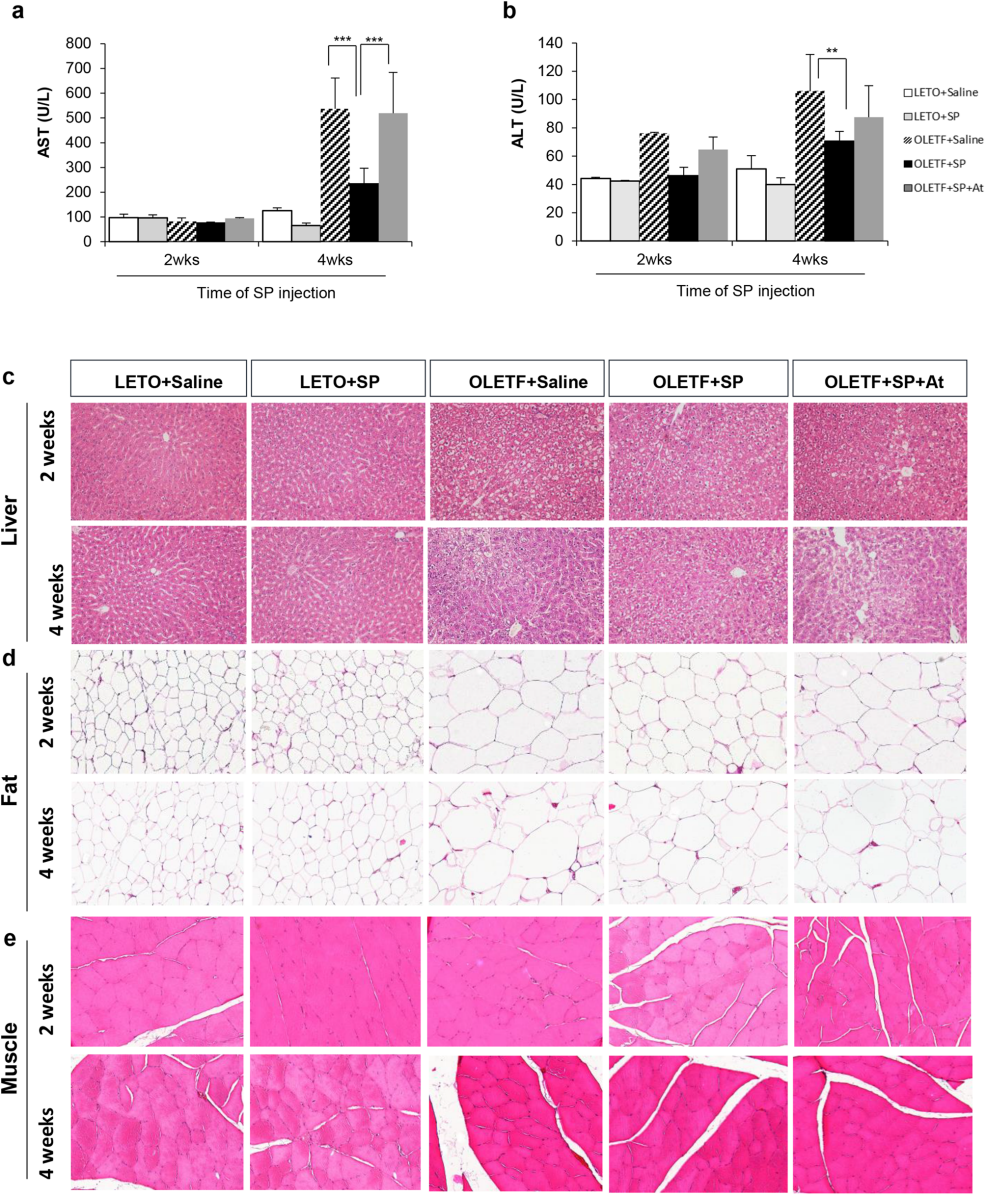
SP blocks the injury of target tissues with insulin resistance. (**a, b**) AST or ALP in serum was determined at 2 and 4 weeks post SP treatment. **c-e,** Histological analysis for target tissue that control glucose level. (**c**) Liver, (**d**) fat and (**e**) muscle. *p* values of less than 0.05 were considered statistically significant ( **p < 0.01, ***p < 0.001). At: NK-1R antagonist. N = 10/group.

The quantification of AST and ALT showed that T2DM-induced hepatic damages were slightly observed at 29 weeks, but reliably developed at 31 weeks (Fig. 4a,b). However, the SP-injected group had lower AST and ALT levels than saline-injected OLETF rats, indicating the lessening of hepatic damages. Histological analysis of hepatic tissue confirmed that the OLETF rat has injured hepatocytes with lipid droplets, and this was mitigated by SP treatment (Fig. 4c).

Comparing with the LETO control, the adipocyte size of fatty tissue was extravagantly enlarged in OLETF rats, and this might be related to the obesity as well as dysfunction of glucose control. The size of the adipocyte decreased in the SP-treated OLETF rat (Fig. 4d), which is likely to contribute to the decrease in the body weight or serum FFA level observed in the SP-injected group. Unlike for the adipose tissue, no specific difference in muscle was detected in all groups (Fig. 4e).

Collectively, the systemic treatment of SP is able to block hepatic injury and adipocyte hypertrophy in OLETF rats, which effect was decreased by NK-1R antagonist. SP-enhanced glucose regulation in OLETF rats may be attributed to the protection of target tissues against diabetic inflammation.

### SP alleviates diabetes-induced systemic inflammation

The diabetic environment provokes pro-inflammatory responses. Notably, obesity is associated with an increase in the pro-inflammatory mediators^28,29^. In recent studies, anti-inflammatory agents were found to prevent fat-induced insulin resistance in rodents, thereby suggesting the involvement of inflammatory pathways in the pathogenesis of fat-induced insulin resistance^30,31^.

**Figure 5:**
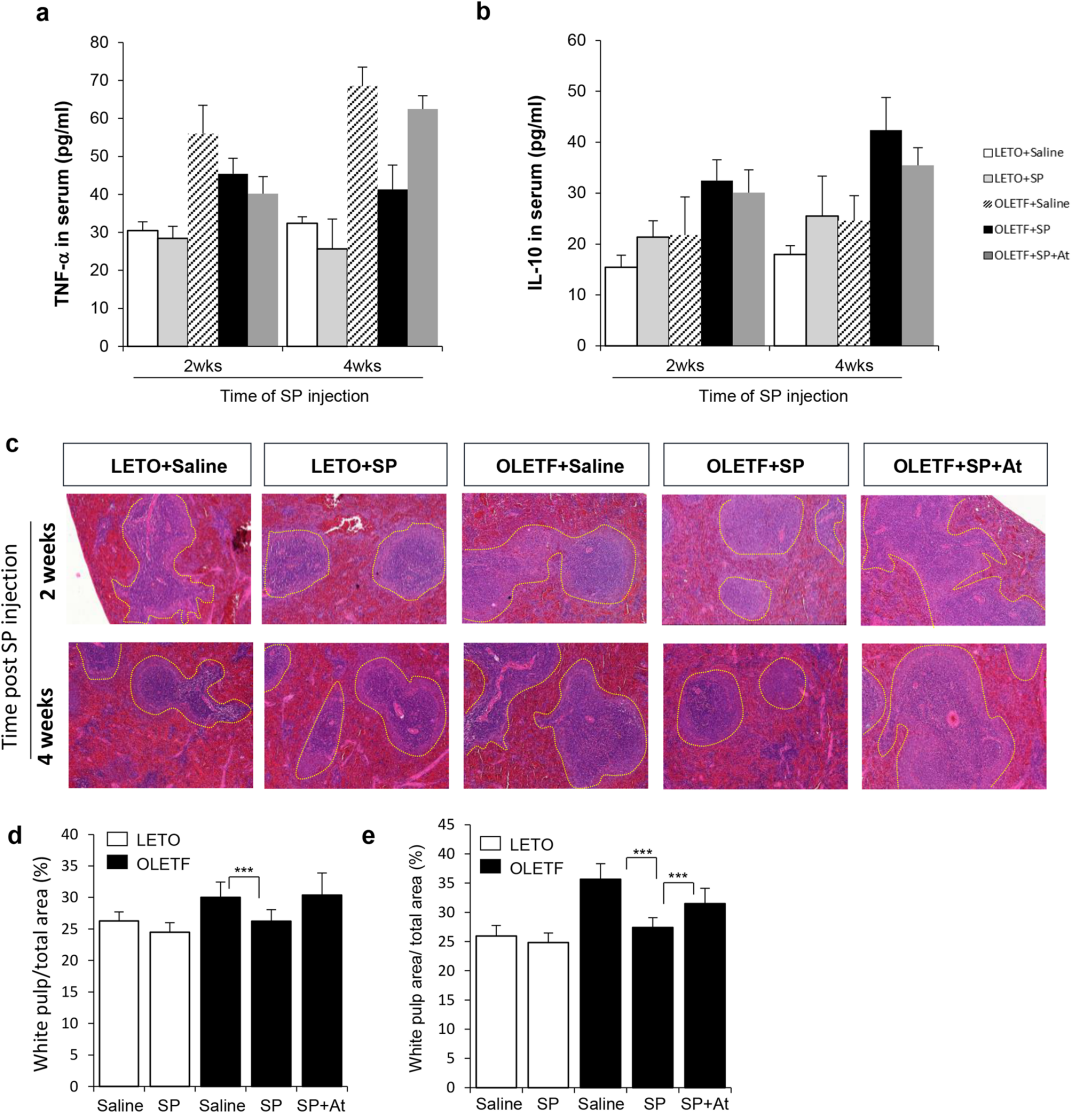
SP suppresses T2DM-induced chronic inflammation. (**a**, **b**) To check the effect of SP on systemic inflammation, serum cytokine, TNF-α (**a**) and IL-10 (**b**) were measured. (**c**) H&E staining for spleen from LETO or OLETF with SP. Yellow dotted line: germinal center. (**d**, **e**) The germinal center was represented as a percentage of total spleen area. 2 week (**d**) and 4 weeks (**e**) after SP injection At: NK-1R antagonist. N = 10/group.

We attempted to check the systemic inflammation in OLETF rats. Saline-injected OLETF rats had a higher serum level of TNF-α than LETO controls. However, SP injection decreased the TNF-α level and elevated IL-10 from 2 weeks post injection. The effect of SP became more evident at 4 weeks after the SP injections (Fig. 5a,b).

The germinal center within spleen is the critical region to produce immune cell during inflammation period and this area is expanded in response to inflammation^32^. Thus, the area of germinal center is used as an indicator to represent the systemic inflammation. The quantification of germinal center in spleen from each group revealed that the OLETF rats had expanded germinal center at 31 weeks, than at 29 weeks. This trend was suppressed by SP treatment (Fig. 5c–e).

These data imply that SP treatment alleviated diabetes-induced chronic inflammation via NK-1R. This might be one of decisive points for the beneficial effect of SP.

### SP improves the bone marrow environment by restoring the pool of stem cells

In T2DM, systemic inflammation and obesity can impair stem cell pool in the bone marrow. A number of reports demonstrated that stem cell pool is decreased in diabetes^7,12^. We previously proved that SP can induce recovery of the bone marrow stem cell pool in diabetes by stimulating the repopulation of stem cells, and SP treatment could inhibit diabetes-induced complications, including ulcer and muscle degeneration, accompanied by immune suppression^12,33^.

**Figure 6:**
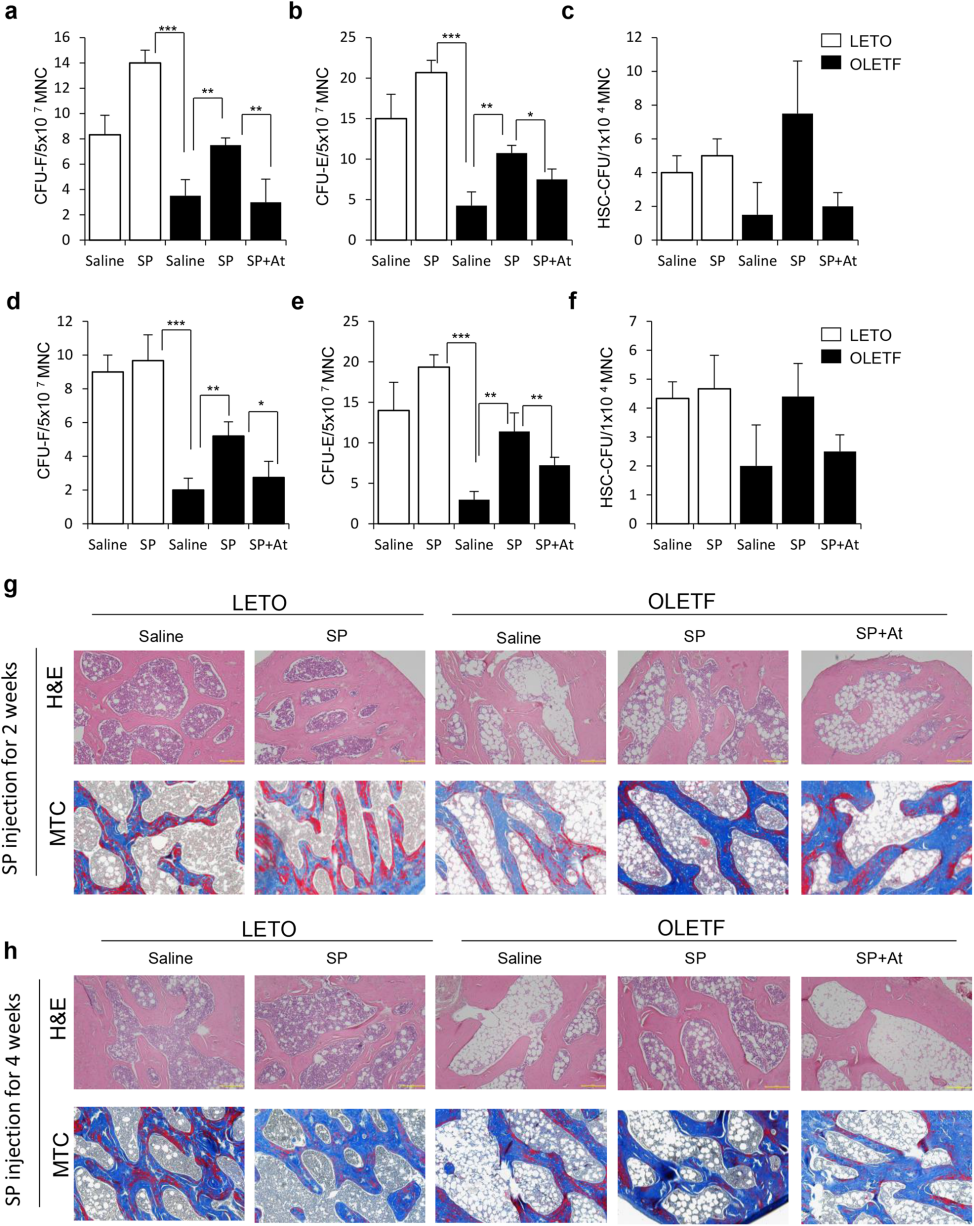
SP treatment rejuvenates bone marrow stem cells and inhibits lipid deposition in the bone marrow. Bone marrow mononuclear cells were cultured and colony-forming ability was assessed. (**a**–**c**) CFU assay (**a**) CFU-F for MSC (**b**) CFU-E for EPC (**c**) and HSC-CFU for HSC at 2 weeks post SP injection. (**d**–**f**) CFU assay (**d**) CFU-F for MSC (**e**) CFU-E for EPC (**f**) and HSC-CFU for HSC at 4 weeks post SP injection. (**g**, **h**) Rat femur was histologically analyzed by H&E staining and Masson trichrome staining. *p* values of less than 0.05 were considered statistically significant (*p < 0.05, **p < 0.01, ***p < 0.001). N = 10/group.

Initially, the stem cell pool in OLETF rats was evaluated. The levels of CFU-F, CFU-E and HSC-CFU were markedly decreased in OLETF rats (Fig. 6a–f). This indicates the lack of regeneration potential by stem cells in OLETF rats. Especially, the impairment of the stem cell pool was already observable at 27 weeks of age before the SP injection (Fig. 1). This worsened at 29 and 31 weeks in OLETF rats, whereby the bone marrow environment was filled with fatty tissue, and was deficient in bone collagen tissue (Fig. 6g,h). Thus, the diabetic condition facilitated the fat accumulation in the bone marrow, creating cellular toxic condition.

Next, we checked whether SP treatment induces the recovery of the stem cell pool in the bone marrow of OLETF rats. The cyclic treatment of SP could restore the stem cell pool by increasing the colony-forming ability of MSC, EPC, and HSCs. The histological analysis corroborated that SP treatment blocked the loss of bone collagen, accompanied by a reduction of fat deposition. This may be attributed to the entire immune modulation by SP.

These data indicate that the pro-inflammatory environment in diabetes may the decrease in the stem cell pool in the bone marrow, and SP treatment is anticipated to be able to rejuvenate the stem cell activity that is impaired by diabetic stress.

### SP injection inhibits progression of diabetes-induced retinal damages

DR is one of the diabetic complications, caused by high blood sugar and microvascular damages in the retina, and it can lead to serious vision loss. We found that the OLETF rat has the impaired glucose regulation, chronic inflammation, deficient stem cell pool, and intra-organ fatty changes, which leads to the expectation of retinopathy in OLETF rats.

**Figure 7:**
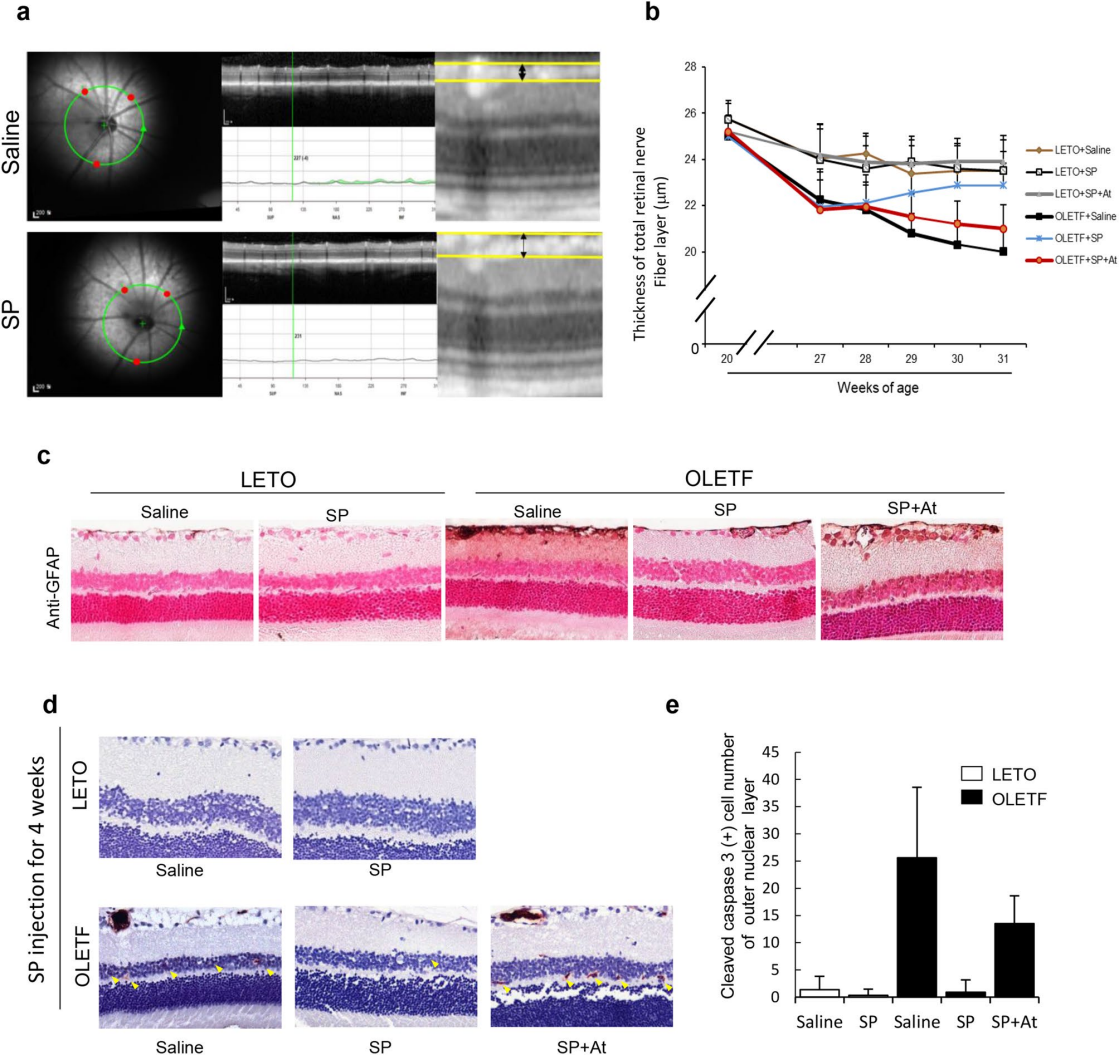
SP can induce the recovery of diabetic retinopathy by suppressing glial activation and cell apoptosis. IR fundus photograph, OCT, and retinal thickness map. The green ring line in the IR fundus photograph indicates the position of the retinal nerve fiber layer (RNFL) thickness circular scan. Red circles are check points for retinal thickness at each AutoRescan. The retinal thickness map automatically shows total retinal thickness (TRT). (**a**) RNFL thickness was manually measured as distance between two yellow boundary lines. (**b**) RNFL thickness from 20 to 31 weeks of age. (**c**) Histology of retina tissue was analyzed for GFAP expression. (**d**, **e**) cleaved caspase 3 + cells were observed by immunostaining and it was quantified. At: NK-1R antagonist. N = 12/group.

To monitor the retinal damage, the total retinal thickness (TRT) and the retinal nerve fiber layer (RNFL) thickness were measured by using SD-OCT from 20 to 31 weeks of age (Fig. 7a). At 27 weeks, the TRT and RNFL differed between the LETO and OLETF rats. Thereafter, the progression of retinopathy rapidly increased. From 28 weeks, the mean TRT of OLETF rats (233.0 ± 3.96 µm) was significantly thinner than those of LETO rats (245.7 ± 4.96 µm), and this difference was maintained through 31 weeks of age. The SP injection did not affect the TRT (Supplementary Fig. 2).

The RNFL thickness was already significantly thinner in OLETF rats (21.9 ± 1.37 µm) than in LETO rats (24.07 ± 1.34 µm) at 27 weeks, and further decreased in a time-dependent manner (Fig. 7b). However, the RNFL thickness was obviously increased by SP treatment (saline: 20.8 ± 0.84; SP: 22.55 ± 0.88 µm, saline vs SP, *p* < 0.001) after 2 weeks post the injection (29 weeks) and this effect was more enhanced at 4 weeks after injection, compared with the saline-injected group (saline, 20.01 ± 0.88 µm, SP:22.88 ± 0.96 µm, saline vs SP, *p* < 0.001). Importantly, the SP injection not only ameliorated the reduction of RNFL thickness, but also increased the RNFL thickness in the diabetic condition.

The effect of SP was evaluated by histological analysis of the retina from each group. Loss of nuclei was observed in both the inner and outer nuclear layers, and decreased thickness was observed in OLETF rats, compared to the LETO controls. However, SP-injected OLETF rats showed a significantly thicker inner retina layer and higher number of nuclei than the saline-injected OLETF (Supplementary Figure 3).

One of the early signs of retinal metabolic stress is the upregulation of GFAP by Müller cells, the main glial cells of the retina. In the normal retina, Müller cells rarely express GFAP. However, GFAP expression increases in response to inflammation due to oxidative stress or injury^34^. Various studies have confirmed the upregulation of GFAP in the diabetic retina^31^. To evaluate glial cell activation in OLETF rats, GFAP immunostaining was undertaken. In LETO rats, the presence of GFAP-positive cells was minimal and restricted to astrocytes. However, OLETF rats had intense GFAP activity, spanning the entire inner retina. The SP injection for 2 weeks did not provide a positive effect on the GFAP expression in the retina (Supplementary Figure 4). However, SP treatment for 4 weeks induced a decrease in GFAP-positive cells in the inner retina in OLETF rats (Fig. 7c).

After diabetes onset, there is an elevated rate of apoptosis is in the outer nuclear layer, with a reduction in photoreceptors. To determine cellular apoptosis, caspase-3^+^ cells were checked by immunohistochemical staining. LETO rats had few apoptotic cells in the retina, which was unaffected by the SP injection. OLETF rats had more caspase-3^+^ apoptotic cells in the outer nuclear layer, and this level was clearly suppressed by SP treatment (Fig. 7d,e).

Collectively, T2DM damages the retinal structure by decreasing the RNFL thickness, activating glial cells, and promoting cell apoptosis. However, SP treatment stably restored the retina, maintaining an intact structure with less apoptotic cells, thereby implying the neuroprotective effect of SP against diabetic stress. Consistent with previous data, SP effect was decreased by NK-1R treatment.

## Discussion

Obesity and T2DM are complexly interrelated and affect multiple organs, leading to insulin resistance. Thus, T2DM necessitates systemic treatment to mitigate the pathological symptoms. However, a stable and effective therapy for T2DM is not available at present.

Chronic obesity increases the circulating FFA levels, which decrease the pancreatic insulin production and destroys the lipid–glucose metabolic balance. Moreover, upregulated FFA induces inflammation and vascular dysfunction. Studies in humans as well as rodents have consistently demonstrated that an experimental elevation in FFA concentrations in healthy subjects reduces the insulin-stimulated glucose uptake^35,36^. Thus, the modulation of obesity and FFA level from adipose tissue may be a critical strategy to prevent the progression of T2DM and its complications.

In this study, OLETF rats showed typical characteristics of T2DM, such as obesity, increased FFA level, systemic inflammation, and suppression of the stem cell pool. These conditions eventually provoked a failure of glucose control and further led to diabetic complications, such as retinal dysfunction. Over time, the pathological symptoms were more strongly apparent.

A previous study found that, at the postnatal 20 weeks in OLETF rats, diabetic retinal damages began to develop and worsened at postnatal 28 weeks, with a reduction of TRT and RNFL^33^. Therefore, we decided to inject SP, biweekly, starting from 27 weeks for 4 weeks at this study and then, evaluated diabetic indicators and the severity of retinopathy.

The systemic injection of SP could decrease the FFA, triglyceride, and leptin levels that are related to obesity from 2 weeks post the injection. SP elevated the adiponectin levels, which are deeply involved in the regulation of blood glucose levels as well as fatty acid breakdown. This condition was likely to alleviate obese-induced dysfunctions. As predicted, the reduction of FFA level by SP could block the pancreatic insulin deficiency, which provides a solution for insulin resistance. The analysis for target tissues responding for insulin resistance revealed that SP injection inhibited hepatic damage, with reduced deposition of lipid droplet and blocked adipocyte enlargement. This effect was evident after 2 weeks of SP and was sustained for 4 weeks. The biochemical markers representative of diabetes could be affected by chronic inflammation. SP injection clearly decreased TNF-α and promoted IL-10 production in the peripheral blood. Moreover, the extended splenic germinal center in OLETF rats tends to be reduced by SP injection.

The SP-induced reduction in inflammation and obese-relating factors was expected to affect the bone marrow microenvironment. Stem cell pools were extremely decreased in the T2DM environment, confirmed by colony-forming ability and histology. Notably, OLETF rats showed high proportions of adipose tissue and reduced collagen expression. This might be caused by chronic inflammation due to obesity. However, SP injection could prevent bone loss and preserved the stem cell pool, which was accompanied by an inhibition of fatty change. This indicates that SP injection is capable of rejuvenating the impaired stem cell function, possibly by stimulating repopulating of stem cell or maintaining immune suppressive condition.

The sustained stress due to chronic inflammation in T2DM promotes the development of diabetic complications. Diabetic retinopathy was developed after 20 weeks, and it deteriorated rapidly after 27 weeks. However, SP treatment could protect the retina by increasing the RNFL, which was observed from 2 weeks post SP injection and reached a level similar to that of LETO rats after 4 weeks of SP injection. Histological analysis suggested that SP could block glial activation and cell apoptosis in the retinal layer. Because SP was injected into the tail vein, SP may not have acted on the retina directly but SP-mediated immune modulation and rejuvenation of stem cells are inferred to influence retinal regeneration in T2DM. Additionally, all effect of SP on diabetic condition and DR was clearly suppressed by NK-1R blocking, indicating that SP effect was occurred via SP-NK-1R binding.

The diabetic condition is known to be rich in neutral endopeptidase that degrades SP. The circulating SP concentration is low in patients with diabetes, compared to non-diabetic individuals^37–39^. Therefore, systemic injection of SP was expected to compensate for the insufficiency of SP in vivo. The application of SP resulted in the diffusion of SP to highly vascularized tissue. Therefore, exogenously injected SP might initially act on immune cells in the blood or perfused organs, and this interaction may create an immunosuppressive condition. However, the mechanism of action of SP for insulin resistance or effect of SP on FFA secretion in adipocyte was unclear, and this should be explored in cells from the liver, fat, and muscle.

Taken together, this study demonstrates the SP can provide anti- iabetic condition and finally attenuate diabetic retinopathy. The safety of SP was corroborated in preclinical study under GLP facility^40,41^. Thus, SP is expected to be a potential agent to prevent progression of retinopathy of T2DM patients.

## Supplementary information

Supplementary Information 1.

## Data Availability

The datasets used in this study are available from the corresponding author on reasonable request.
