# Supplementary material for: SP prevents T2DM complications by immunomodulation
Source: Sci Rep. 2020 Oct 7;10:16753. doi: 10.1038/s41598-020-73994-1 (PMC7541476; doi:10.1038/s41598-020-73994-1)
Supplement: Supplementary file 1 — Supplementary Information 1. [file 41598_2020_73994_MOESM1_ESM.pdf]

SP prevents T2DM complications by immunomodulation

Sang Min Baek†, Kiyoung Kim†, Suna Kim, Youngsook Son, Hyun Sook Hong*, Seung-Young Yu*

***Correspondence:** Hyun Sook Hong, Ph.D.
Department of Biomedical Science and Technology, Graduate School,
Kyung Hee University, Phone: +82-2-958-1828/ E-mail: hshong@khu.ac.kr

Supplementary Figure 1

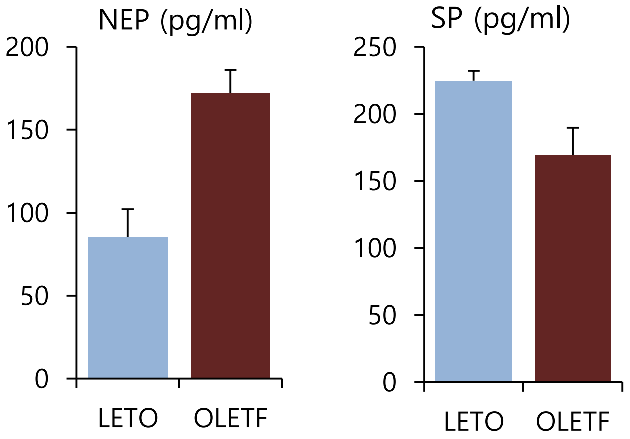

Supplementary Figure 1. The analysis of NEP and SP in serum of LETO and OLETF at 27weeks

Blood was collected with syringe including aprotinin (0.014TIU/ml) and centrifuged at 10000rpm for 5 min for serum isolation. NEP (Neutral endopeptidase) and SP level in serum was quantified by ELISA

Supplementary Figure 2

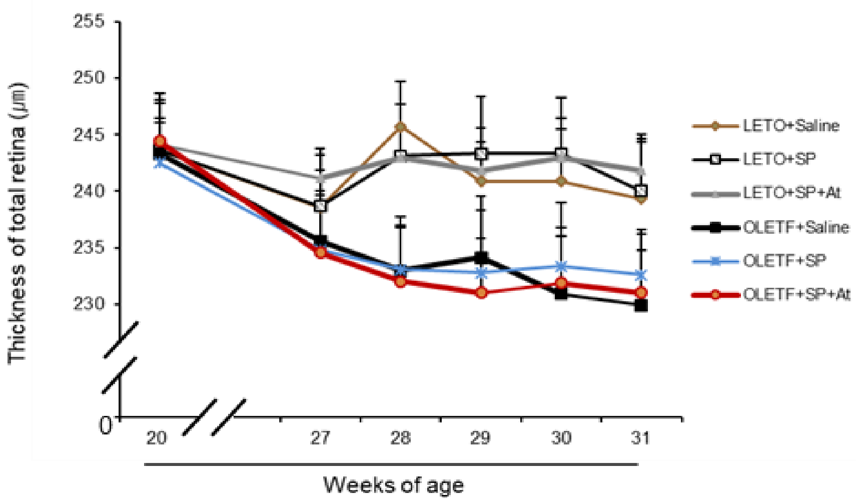

Supplementary Figure 2.The analysis of total retina thickness
Total retina thickness was monitored from postnatal 20 weeks to 31 weeks.

Supplementary Figure 3

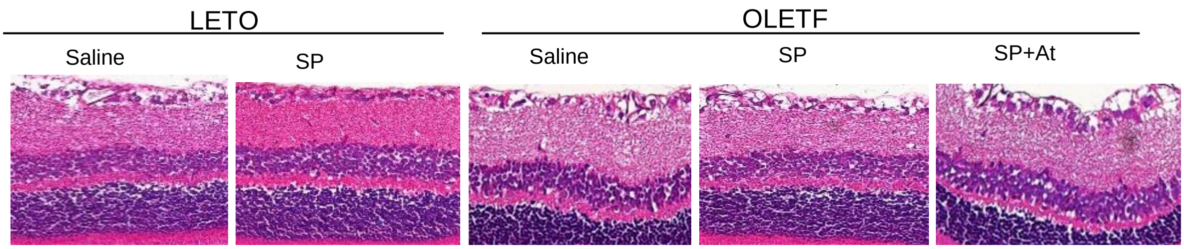

Supplementary Figure 3. The effect of SP on retina structure
SP was injected for 4 weeks and then, H&E staining was performed to observe retina structure

Supplementary Figure 4

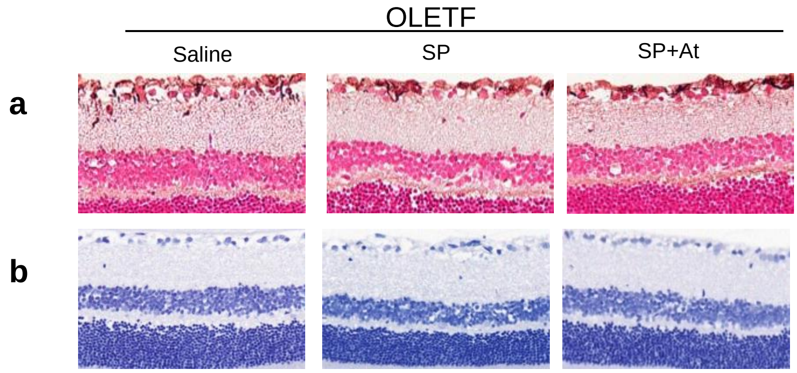

Supplementary Figure 4. The effect of SP on retina structure
SP was injected for 2 weeks from postnatal 27 weeks and then, GFAP expression (**a**) and cleaved caspase 3+ cell (**b**) were checked by immunohistochemically staining
